# Genome Sequence of a Multidrug-Resistant Strain of *Klebsiella pneumoniae*, BAMC 07-18, Isolated from a Combat Injury Wound

**DOI:** 10.1128/genomeA.01230-14

**Published:** 2014-11-26

**Authors:** Tricia A. Van Laar, Tsute Chen, Brandon M. Childers, Ping Chen, Johnathan J. Abercrombie, Kai P. Leung

**Affiliations:** a Microbiology Branch, U.S. Army Dental and Trauma Research Detachment, Institute of Surgical Research, JBSA Fort Sam Houston, San Antonio, Texas, USA; b The Forsyth Institute, Cambridge, Massachusetts, USA

## Abstract

*Klebsiella pneumoniae* is an important infectious agent of surgical sites and combat wounds. Antibiotic resistance and tolerance are common impediments to the healing of chronic infections. Here, we report the genome sequence of a highly multidrug-resistant strain of *K. pneumoniae*, BAMC 07-18, isolated from a combat wound of a soldier.

## GENOME ANNOUNCEMENT

*Klebsiella pneumoniae*, a Gram-negative bacterium, is commonly found in the soil and as a component of the normal human flora (1). However, *K. pneumoniae* has been increasingly implicated as a causative agent of nosocomial and/or chronic infections (2). Multidrug-resistant (MDR) strains of *K. pneumoniae* are also one of the most common pathogens isolated from infections in soldiers wounded in combat (3–5). The tendency of these strains to form biofilms on biotic and abiotic surfaces, including catheters and other medical devices, is a contributing factor to their antibiotic resistance (6). *K. pneumoniae* BAMC 07-18 (kindly provided by Clinton Murray of the San Antonio Military Medical Center, Fort Sam Houston, San Antonio, TX) is a biofilm-forming MDR strain isolated from a patient at the San Antonio Military Medical Center (SAMMC). This strain was highly resistant to many antimicrobials, including azithromycin, ceftazidime, chloramphenicol, and tetracycline; however, BAMC 07-18 is sensitive to imipenem, a carbapenem, both *in vivo* and *in vitro* (7). High doses of imipenem are still unable to completely clear biofilm infections, though it leads to significant reductions in viability and alterations in morphology (7), leading us to question the genetic mechanisms of the pleiotropic effects of imipenem against this carbapenem-sensitive strain of *K. pneumoniae*.

As a first step to understanding these effects, we sequenced the genome of *K. pneumoniae* BAMC 07-18. *De novo* genomic sequencing service was provided by BGI Tech Solutions Co., Ltd. (Cambridge, MA, USA) using the Illumina HiSeq 2000 platform. A total of 602 Mb of data was produced for BAMC 07-18 from the 500-bp library, 604 Mb of data from the 2,000-bp library, and 351 Mb of data from the 6,000-bp library. The raw sequence data were quality filtered and then assembled using the SOAP*denovo* software (8).

The preliminary total assembled genome size was 5.5 Mb, consisting of a 5.0-Mb chromosome and 8 contigs ranging from 0.5 to 447.6 kb, with a G+C content of 57.20%, without low-coverage regions.

An analysis of the genome sequence revealed the presence of many genes for antibiotic resistance, such as extended-spectrum β-lactamases (CTX-M, SHV, and TEM), polymyxin, tetracycline, and chloramphenicol, with a lack of any genes implicated in carbapenem resistance (9–12). We also found common virulence factors, such as genes necessary for biofilm and capsule formation, adhesion, and iron sequestration (13–19). The use of this genomic sequence as a reference for RNA sequencing analysis (RNA-seq) will allow us to explore the pleiotropic effects of carbapenems on *K. pneumoniae* biofilms and provide novel opportunities to exploit the overall fitness of *K. pneumoniae* under carbapenem stress.

### Nucleotide sequence accession number.

This genome sequence is deposited in GenBank under the accession no. JRQE00000000.
